# Microdissection testicular sperm extraction–intracytoplasmic sperm injection strategy in patients with Klinefelter syndrome: synchronous or asynchronous?

**DOI:** 10.3389/fendo.2025.1585818

**Published:** 2025-06-30

**Authors:** Ke Feng, Jin-Wei Wang, Yan-Qing Xia, Xiao-Wei Qu, Feng Wan, Bo Zhang, Cui-Lian Zhang, Hai-Bin Guo, Lei-Lei Feng, Ying-Hong Fang

**Affiliations:** ^1^ Department of Reproductive Medicine Center, Henan University People’s Hospital, Zhengzhou, China; ^2^ Reproductive Medicine Center, Henan Provincial People’s Hospital, Zhengzhou, China; ^3^ Department of Reproductive Medicine Center, Zhengzhou University People’s Hospital, Zhengzhou, China

**Keywords:** Klinefelter syndrome(KS), microdissection testicular sperm extraction(mTESE), intracytoplasmic sperm injection(ICSI), synchronous, asynchronous

## Abstract

**Objective:**

The objective of this study was to compare the clinical outcomes of intracytoplasmic sperm injection (ICSI) with fresh versus cryopreserved sperm retrieved via microdissection testicular sperm extraction (mTESE) in patients with Klinefelter syndrome (KS).

**Design:**

A retrospective cohort study was conducted.

**Setting:**

This study was performed at the Reproductive Medicine Center of Henan Provincial People’s Hospital.

**Participants:**

A total of 260 men with KS, including 5 patients with mosaic KS, underwent mTESE,124 of whom successfully provided sperm. These patients were divided into synchronous (fresh sperm) and asynchronous (cryopreserved sperm) groups for ICSI treatment.

**Interventions:**

Fresh or cryopreserved sperm were used in the ICSI cycles.

**Main outcome measures:**

The primary outcomes were the clinical pregnancy rate, live birth rate, and miscarriage rate. The secondary outcomes were two pronuclei (2PN) embryos, available embryos, and the blastocyst formation rate. The number of oocytes retrieved and metaphase II (MII) oocytes were considered female-related indicators and treated as potential confounding variables in the multivariate analyses, given their influence on embryo development and pregnancy outcomes.

**Results:**

A total of 260 KS patients underwent mTESE, with the successful retrieval of sperm suitable for ICSI in assisted reproduction from 124 (47.7%). Among these, 73 had their sperm cryopreserved at low temperature before ICSI, while 51 had their fresh sperm directly used for ICSI. The analysis of 170 treatment cycles revealed no significant differences in baseline characteristics (infertility duration, body mass index (BMI), follicle stimulating hormone (FSH), luteinizing hormone (LH), testosterone (T)) between the groups. Compared with the asynchronous group, the synchronous group had more oocytes retrieved, MII oocytes, gestational sacs, and good-quality embryos. However, there were no significant differences in 2PN embryos, 2PN fertilization rates, total embryos, available embryos, good-quality embryo rates, clinical pregnancy rates, live birth rates, or miscarriage rates between the two groups. LASSO regression and ROC curve analysis demonstrated the limited ability of differential indicators to predict pregnancy outcomes.

**Conclusions:**

In KS patients undergoing ICSI, the use of fresh or cryopreserved testicular sperm did not significantly affect pregnancy outcomes. While fresh sperm have advantages in improving certain laboratory parameters, their overall ability to predict pregnancy outcomes is limited.

## 1 Introduction

Klinefelter syndrome (KS) is one of the most common genetic causes of male infertility and a major pathological type of nonobstructive azoospermia (NOA). It is typically caused by an additional X chromosome (47, XXY) and has a reported prevalence of 1:500–1,000 in the male population, accounting for an estimated 1%–2% of male infertility cases (1). Approximately 80%-90% of KS patients exhibit the typical 47,XXY karyotype, known as KS, whereas the remaining 10%-20% present mosaicism, such as 46,XY/47,XXY, or higher-order chromosomal abnormalities (e.g., 48,XXXY, 49,XXXXY) (2, 3). Men with KS typically present with symptoms such as gynecomastia, small testes, azoospermia, and elevated gonadotropin levels. Historically, the majority of KS patients, owing to NOA, were considered incapable of reproduction and had to rely on donor sperm for assisted reproduction. However, advancements in assisted reproductive technologies have opened new pathways to parenthood for KS patients. Microdissection testicular sperm extraction (mTESE) combined with ICSI has enabled successful sperm retrieval and fertilization in this population (4–6).Despite widespread pathology in the seminiferous tubules of KS patients, mTESE allows for the precise localization and extraction of sperm, often yielding sufficient quantities for ICSI and improving the chances of conception (7, 8). Recent studies have investigated the impact of different etiologies on the sperm retrieval rate (SRR) in NOA patients undergoing mTESE and the subsequent treatment outcomes of ICSI. The findings suggest that the SRR correlates with the etiology and histological categories of NOA, whereas reproductive outcomes are more closely linked to good-quality embryo rates and the age of the female partner (9). In practical applications of mTESE-ICSI, the timing of sperm retrieval can present challenges. If fresh sperm cannot be used on the day of ICSI, cryopreservation becomes necessary. To mitigate the risk of failed sperm retrieval, some couples opt for mTESE and sperm freezing before ovarian stimulation and egg retrieval, subsequently using the cryopreserved sperm for ICSI. Oates et al. were the first to describe the pregnancy-related results of using cryopreserved sperm for ICSI (10, 11). Despite these findings, most studies have primarily focused on synchronous and asynchronous procedures in NOA patients, with limited attention given to KS patients as a distinct group. To bridge this gap, we conducted a retrospective analysis focusing on KS patients, comparing the clinical outcomes of the use of fresh versus cryopreserved sperm in mTESE-ICSI.

## 2 Methods

This retrospective analysis was conducted between December 2016 and June 2024 at the Reproductive Medicine Center of Henan Provincial People’s Hospital. This study focused on KS patients, identified according to the World Health Organization (WHO) definition of azoospermia, confirmed by the absence of sperm in at least two semen analyses. All enrolled patients were diagnosed with KS, including 5 patients with mosaic KS. They were diagnosed based on clinical presentation and confirmed by peripheral blood karyotype analysis. The diagnosis required the presence of a 47,XXY karyotype, or mosaic variants such as 46,XY/47,XXY, in at least 20 metaphase spreads. Before undergoing mTESE, all participants completed detailed physical examinations and hormonal evaluations. These assessments included measurements of follicle-stimulating hormone (FSH), luteinizing hormone (LH), prolactin (PRL), estradiol (E2), and testosterone (T). Testicular volume was assessed through both physical examination and ultrasound imaging. The inclusion criteria required male participants to be diagnosed with Klinefelter syndrome, aged between 20 and 35 years, and have a body mass index (BMI) ranging from 18 to 30 kg/m². Female partners were excluded if they had ovulatory dysfunction, hormonal imbalances, infertility, tubal obstruction, endometriosis, or other factors known to affect fertility. The exclusion criteria included patients with chronic diseases such as diabetes, hypertension, heart disease, postorchiectomy, mumps orchitis with testicular inflammation, hypogonadotropic hypogonadism, hypergonadotropic hypogonadism, or obstructive azoospermia, as well as couples where female infertility was the primary cause of infertility. From December 2016 to June 2024, a total of 260 KS patients underwent mTESE treatment, with sperm successfully retrieved from 124 patients. The study included 170 treatment cycles. The patients were divided into two groups according to the type of sperm used for ICSI (fresh or cryopreserved): synchronous group (using fresh oocytes and fresh sperm, 51 patients, 67 cycles);and asynchronous group (using fresh oocytes and cryopreserved sperm, 73 patients, 103 cycles). The study was approved by the Ethics Committee of the Reproductive Medicine Center, Henan Provincial People’s Hospital (Approval No. [SYSZ-LL-2019110401]), and all participants provided written informed consent. All participants were followed up until delivery.

### 2.1 Micro-TESE surgery and sperm retrieval

In this study, mTESE was performed under general anesthesia. After strict disinfection and draping, a longitudinal incision was made along the midline of the scrotum, and the skin, tunica, and spermatic cord were opened sequentially to expose and extrude one side of the testis and epididymis. Under a surgical microscope (Carl Zeiss S88), a fine scalpel was used to make an incision along the equator of the testis, and slight tension was applied to fully expose the testicular tissue. The magnification was adjusted to 10–25 times, and the seminiferous tubules on the exposed surface were examined. Full, thickened, and well-formed seminiferous tubules were selected and sent to the embryology laboratory for microscopic examination. The presence of sperm was checked under an inverted microscope (×400), and if usable sperm were found, the surgery was concluded. If no sperm were found, the tunica albuginea of the testis was bluntly separated along the equatorial incision, and suitable seminiferous tubules were selected for further examination. If sperm were still not found, the testicular parenchyma was progressively dissected in layers, and full, thickened seminiferous tubules were selected for further inspection. Special care was taken to protect the blood vessels beneath the white membrane of the testicle and within the testicular parenchyma. If no sperm were found on one side of the testis, the opposite testis was opened and treated via the same method. After surgery, the white membrane was sutured intermittently with 5–0 absorbable sutures, the testis was repositioned into the tunica, and continuous sutures were applied to the scrotal skin. For patients in whom no sperm were found during the procedure, the extracted seminiferous tubules were placed in culture medium overnight. The next morning, the tissues were reexamined, and the results were confirmed by two independent reviewers.

### 2.2 Fresh sperm and sperm cryopreservation

After fresh sperm were obtained through testicular sperm extraction under a microscope, they were directly used for ICSI treatment on the same day if fresh oocytes were available. If oocytes could not be obtained on the same day, the sperm were cryopreserved. The freezing process was as follows: First, the testicular suspension was thoroughly mixed with sperm freezing solution (Vitrolife) at a 1:1 ratio via a 2 ml pipette for dilution. The mixture was then left to stand at room temperature for 10 minutes. Afterward, the sample was placed on a polystyrene foam board and rapidly cooled in a liquid nitrogen bath, where it remained for 30 minutes. The cryopreserved sperm samples were subsequently stored in liquid nitrogen until use.

### 2.3 Sperm thawing and processing

When the sperm were used, the cryopreserved sperm sample was removed from liquid nitrogen and placed in a 37°C incubator for 15 minutes. The sample was then washed with culture medium and centrifuged at 300 × g for 10 minutes, and the process was repeated twice (using a BY-160A centrifuge, Beijing, China) to remove the cryoprotectants. The sperm sample was then resuspended in culture medium (Vitrolife) in preparation for the next step of the ICSI procedure.

### 2.4 ICSI assisted reproductive treatment

A total of 51 female patients underwent synchronized controlled ovarian stimulation and directly received ICSI treatment after successful sperm retrieval. In contrast, 73 female patients who did not undergo synchronized ovarian stimulation had their partner’s sperm cryopreserved using microquantity sperm freezing techniques after successful retrieval, and ICSI treatment was performed after sperm thawing.

### 2.5 Ovarian stimulation and oocyte retrieved

In couples undergoing mTESE and ICSI treatment, the female partner usually requires ovarian stimulation. Ovarian stimulation and egg retrieval are essential components of assisted reproductive technology. To increase the number of mature eggs, ovarian hyperstimulation is applied to promote the growth of multiple follicles simultaneously. The medications commonly used for ovarian stimulation include GnRH agonists (GnRH-a), GnRH antagonists (GnRH-ant), and follicle-stimulating hormone (FSH). The choice of stimulation protocol—such as a long protocol, short protocol, or antagonist protocol—is tailored to the patient’s ovarian reserve. Follicular development, including estradiol and luteinizing hormone levels, is closely monitored through ultrasound imaging and hormone level assessments. When the dominant follicle reaches a diameter of 18–20 mm, a final maturation trigger is administered, typically human chorionic gonadotropin (hCG) or GnRH-a. Egg retrieval is scheduled approximately 34–36 hours later. During the procedure, a transvaginal ultrasound probe is used to visualize the ovaries and follicles. The probe is carefully adjusted to align with the target follicles. A fine needle, connected to a suction device, is inserted through the vaginal wall to aspirate the follicular fluid. This mixture, which contains eggs and granulosa cells, is collected in sterile tubes. The process is completed on one ovary before proceeding to the other to retrieve all mature follicles. The collected follicular fluid is promptly sent to the laboratory, where eggs are identified under a microscope. After identification, the eggs are placed in culture media and transferred to an incubator for further development. Strict aseptic techniques and short-acting anesthesia are employed during the procedure to ensure patient safety and comfort. Postoperative monitoring is crucial for detecting and managing potential complications such as ovarian hyperstimulation syndrome (OHSS), bleeding, or infection.

### 2.6 Criteria for sperm selection during ICSI

Regardless of whether fresh or cryopreserved sperm is used in ICSI treatment, the key criteria for sperm selection focus on motility and morphology to ensure fertilization success and embryo quality. Priority is given to sperm with an oval and smooth head, a symmetrical midpiece, and an intact tail, while avoiding vacuolated or morphologically abnormal sperm whenever possible. To ensure reliable results, all procedures were conducted under controlled laboratory conditions, following strict protocols at each stage.

### 2.7 Definition

ICSI was performed according to standard operating procedures. Fertilization was assessed by observing the presence of pronuclei (2PN) and two polar bodies 17 to 19 hours postfertilization. Embryo transfer was typically carried out on the third day after oocyte retrieval. The embryos were graded via the Society for Assisted Reproductive Technology (SART) scoring system, and only the best quality embryos were selected for transfer. The number of embryos transferred was usually limited to two to reduce the risk of multiple pregnancies. “Successful sperm retrieval” was defined as the identification of at least one motile or morphologically normal spermatozoon suitable for ICSI on the day of surgery.

### 2.8 Statistical analysis

The data are presented as the means ± standard deviations (SDs) or interquartile ranges, and categorical variables are expressed as percentages. For continuous variables, if the data were normally distributed and had equal variances, one-way analysis of variance (ANOVA) was used for inter-group comparisons; otherwise, the Kruskal–Wallis test was applied. Differences in categorical variables were analyzed via the chi-square test or Fisher’s exact test, depending on whether the expected frequency met the assumptions for the chi-square test. All the statistical analyses were performed using IBM SPSS Statistics software (version 25.0, IBM Corp., Armonk, NY, USA). A p-value of less than 0.05 was considered statistically significant. Logistic regression analysis and LASSO regression were performed to construct a pregnancy prediction model based on the differential indicators, followed by cross-validation. The number of oocytes retrieved and MII oocytes, although not study outcomes, were included in regression models as female-related confounding variables due to their known influence on embryo development and pregnancy success. The nomogram was created via the regression modeling strategy package in R software (version 4.2.1). The ROC curve was plotted using the ‘pROC’ package, and the area under the curve (AUC) and 95% confidence interval (CI) were calculated.

## 3 Results

### 3.1 Results of testicular sperm ICSI

Among the 260 KS patients who underwent mTESE, spermatozoa suitable for ICSI were successfully retrieved from 124 patients. Among these patients, 73 opted for sperm freezing prior to ovarian stimulation. All cryopreserved samples met the ICSI quality criteria. Sperm retrieval was successful in 47.7% of the initial cohort. No significant procedural complications occurred.

### 3.2 Baseline characteristics of participants

There were no significant differences in the ages of the male and female participants between the two groups (p > 0.05), and no statistically significant differences were observed in other patient characteristics (such as infertility duration, BMI, or hormone levels). The baseline characteristics of all patients are presented in **Table 1**.

**Table 1:** Patient baseline characteristics and comparison of age, testicular volume and reproductive hormone between two groups.

Character	Synchronous group (n=51)	Asynchronous group (n=73)	P
Age (year)			
Male Female Infertile time (year) BMI (kg m−2) Male Female Height (cm) Male Female Weight (kg) Male Female FSH (mIU ml−1) LH (mIU ml−1) PRL (ng/mL) E2 (pg/mL) T (pg/ml) Testicular volume (ml)	29 (25, 32) 27 (25, 30.5) 2 (1, 3) 24.99 ± 4.2 23.63 (21.18, 26.88) 178 (175, 180) 160 (158, 164.5) 80 (70, 85) 60 (54.5, 69.5) 35.2 (28.09, 43.52) 23.28 ± 6.77 12.36 (9.14, 17.38) 22.29 ± 8.74 1.77 (1.23, 2.2) 2 (2,4)	28 (25, 30) 27 (25, 30) 2 (1.5, 3) 23.68 ± 3.79 22.49 (20.5, 25.39) 178 (174, 180) 160 (158, 163) 75 (69.5, 83) 60 (52.5, 65) 36.82 (29, 46.37) 22.48 ± 7.97 12.52 (10.35, 18.84) 24.51 ± 7.61 2.05 (1.57, 2.55) 2 (2,4)	0.502 0.731 0.103 0.079 0.240 0.744 0.636 0.076 0.348 0.401 0.548 0.427 0.145 0.055 0.77

FSH, Follicle-Stimulating Hormone; LH, Luteinizing Hormone; PRL, Prolactin; E2, Estradiol; T, Testosterone.

### 3.3 Comparison of female-related parameters laboratory indicators, and clinical outcomes between the synchronous and asynchronous groups

The synchronous group demonstrated significantly higher values in female-related parameters (i.e., oocytes retrieved and MII oocytes), as well as in the number of gestational sacs and good-quality embryos, compared to the asynchronous group (p < 0.05). However, no significant differences in the number of 2PN embryos, 2PN fertilization rate, total number of embryos, number of available embryos, or good-quality embryo rate, were detected between the two groups, as shown in **Table 2**. Additionally, the clinical pregnancy rates, live birth rates, and miscarriage rates did not significantly differ between synchronous group and asynchronous group (all p > 0.05), as shown in **Table 3**.

**Table 2:** Comparison of laboratory indicators and female-related variables between two groups.

Parameters	Synchronous group (n=51)	Asynchronous group (n=73)	P
Cycles (n) Oocytes (n)	67 12.27 ± 3.09	103 10.75 ± 3.65	0.016*
MII oocytes (n)	10 (8, 12.5)	8 (6, 11)	0.010*
2PN (n) 2PNFertilization rate (%)	7 (4, 10) 0.75 (0.54,0.90)	5 (4, 9) 0.7 (0.5,0.8)	0.174 0.143
Total Number of Embryos (n) Good-quality embryos available embryos Good-quality embryo rate (%)	7 (3.5, 10) 3 (1,5.5) 5 (2, 7.5) 0.48 ± 0.28	5 (4, 9) 2 (0,4) 3 (1,6) 0.36 (0,0.67)	0.247 0.030* 0.167 0.144
Gestational sac	1(0,1)	0 (0,1)	0.029*

*P<0.05, Synchronous group versus asynchronous group. Date are presented as mean ± s.d. or IQR, synchronous group: fresh sperm; asynchronous group: cryopreserved sperm. MII oocytes, metaphase II oocytes; 2PN, 2 pronuclei; s.d., standard deviation.

**Table 3:** Pregnancy Outcomes Between Synchronous and Asynchronous Groups.

Parameters	Synchronous group(n=51)	Asynchronous group(n=73)	P
Cycles (n) Clinical pregnancy rate, (%)	67 47.7	103 39.3	0.068
Implantation rate, (%) Live birth rate, (%) Miscarriage rate, (%) Birth defects (n)	38 39 7 0	36 30 9 0	0.440 0.391 0.887

Synchronous group: fresh sperm; asynchronous group: cryopreserved sperm.

### 3.4 Construction and validation of the prediction model

**Figure 1:**
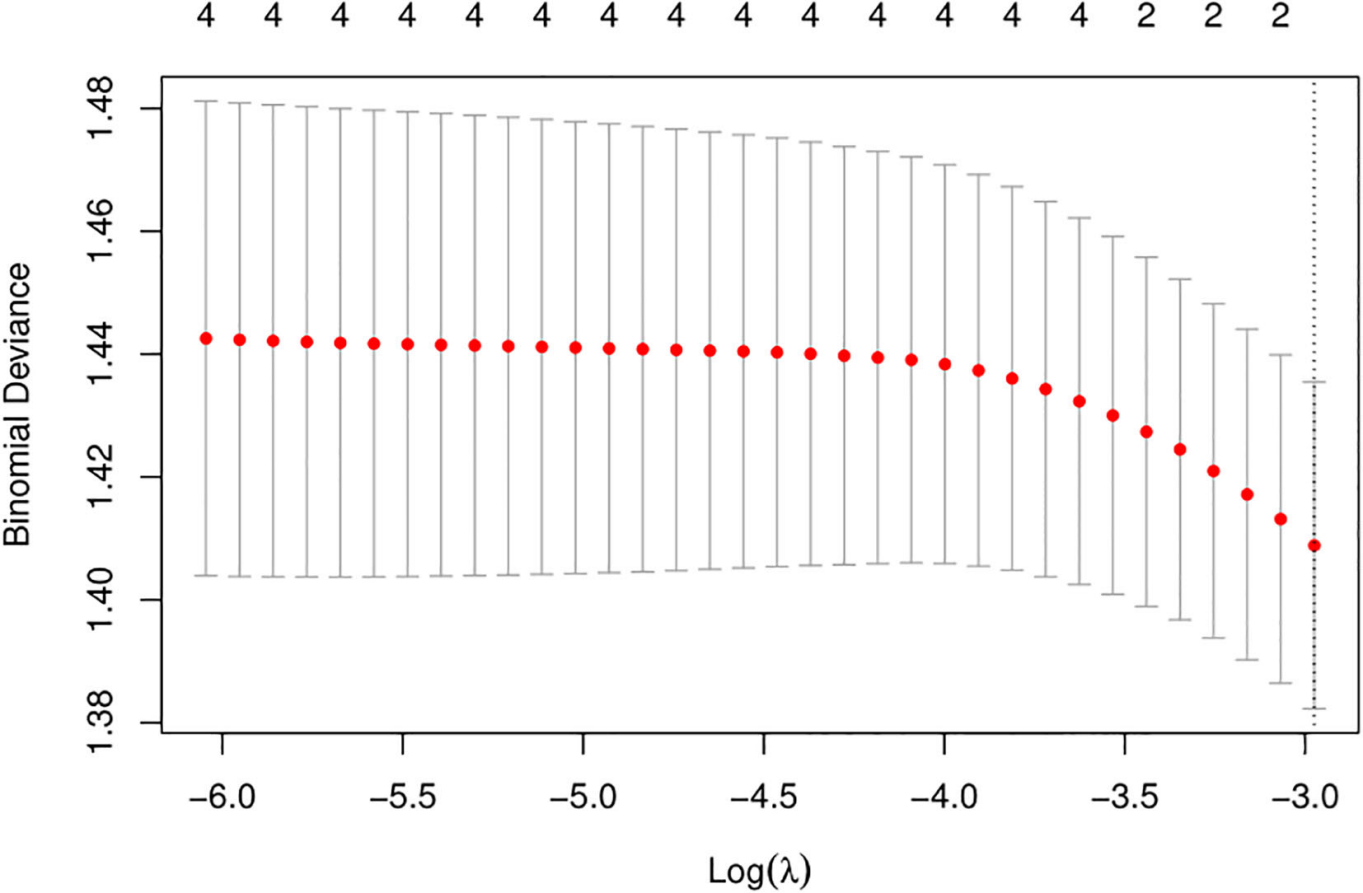
LASSO variable selection. LASSO regression is used to select the most important features. It achieves sparsity by adding a penalty term (L1 regularization) to the optimization objective, forcing many of the feature weights in the coefficient vector to become zero. By selecting the features corresponding to the non-zero coefficients, the most predictive features are identified. This process helps to select the target variable, simplifying the model and improving its generalization ability. Depending on different threshold values, the range of valid variables selected by the LASSO algorithm is 0.

**Figure 2:**
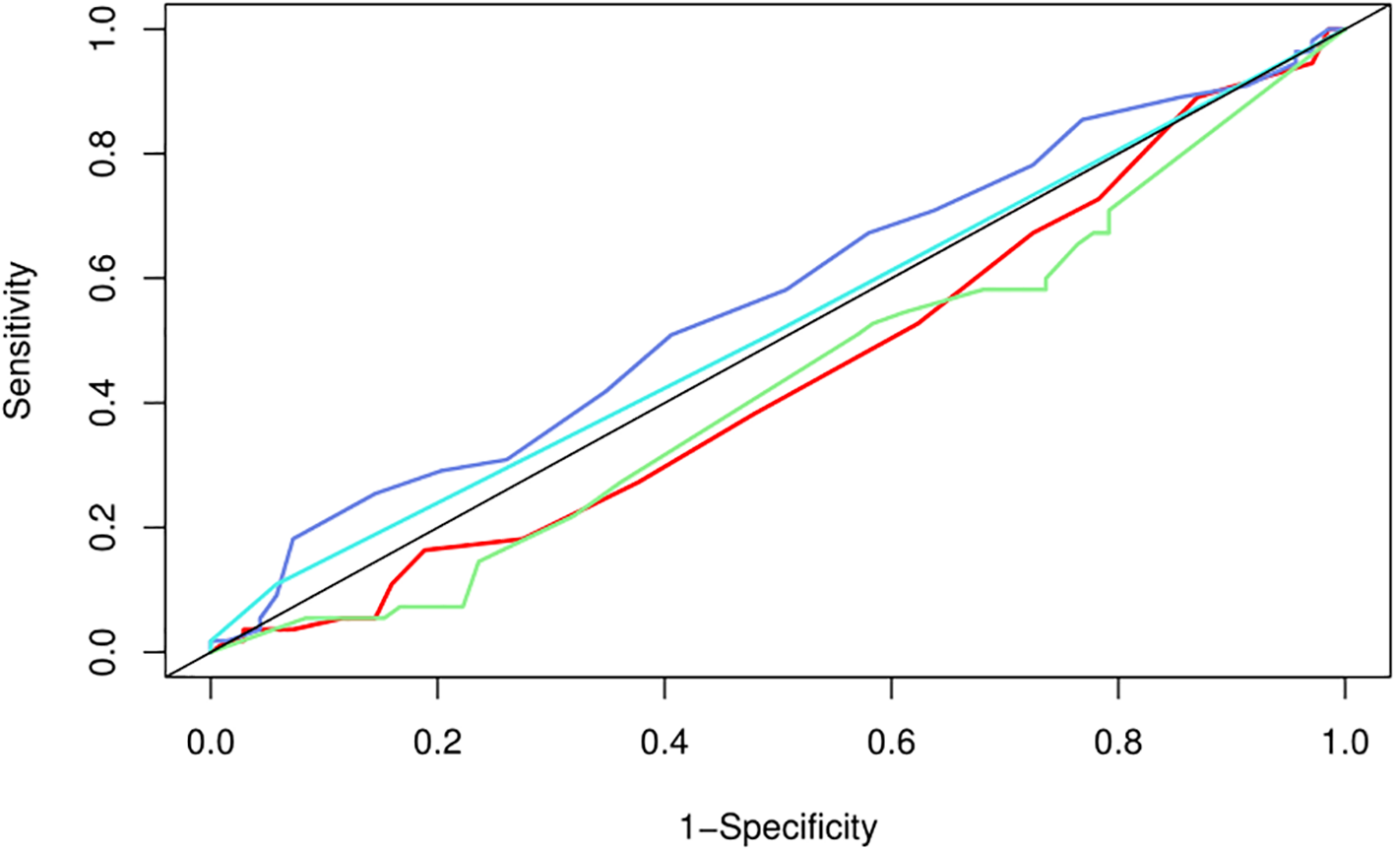
ROC curve for pregnancy prediction model. The ROC (Receiver Operating Characteristic) curve of the nomogram for the pregnancy prediction model. The solid line represents the ability of the prediction model to distinguish between pregnancy and non-pregnancy. The closer the curve is to the point (0,1), the better the performance of the model. The curves in different colors represent various indicators: red for M2 oocytes, purple for oocytes, blue for gestational sac, and green for good-quality embryos.

We used LASSO regression feature selection to choose four factors (**Figure 1**). However, no significant variables were identified, suggesting that the differential indicators have limited ability to predict pregnancy outcomes. Additionally, we further plotted the ROC and calibration curves to assess the model (**Figure 2**). The AUC values and 95% CI for the four differential indicators are shown in **Table 4**. Among them, the AUC values for good-quality embryos and MII oocytes were less than 0.5, indicating poor predictive ability, whereas the AUC values for oocytes and gestational sac were between 0.5 and 0.6, suggesting the model has some classification capability but still has room for improvement, with an overall moderate predictive performance.

**Table 4:** The specific AUC value and 95% CI of the difference indicators.

Difference indicators	AUC	95%CI
Oocytes	0.564	0.461-0.666
Good-quality embryos	0.476	0.376-0.577
Gestational sac	0.520	0.427-0.613
MII oocytes	0.441	0.340-0.542

### 3.5 Pregnancy and pediatric follow-up

A total of 50 healthy children were born as a result of this study. Due to being lost to follow-up, five patients were unable to provide complete follow-up data and provided only written information regarding the delivery and health status of their newborns. The pregnancy outcomes for all fetuses are summarized in **Table 5**.

**Table 5:** Comparison of fetal indicators between the two groups.

Parameters	Synchronous group(n=24)	Asynchronous group(n=26)	P
Gender Male Female Fetal Count Single Fetus (n)	14 10 16	14 12 16	0.740 0.713 0.768
Double Fetus (n) Height (cm) Weight (g)	4 50 (50, 50) 3195.24 ± 490.14	5 50 (49, 50) 3169.26 ± 793.69	0.768 0.399 0.862
Health Condition Fair Poor Good	9 1 14	7 0 19	0.603 0.293 0.461

Date are presented as mean ± s.d or IQR. Synchronous group: fresh sperm; asynchronous group: cryopreserved sperm.

## 4 Discussion

KS is a primary subtype of NOA, and testicular pathology in KS often involves damage to seminiferous tubules, fibrosis, and interstitial changes, all of which contribute to infertility (12). In NOA patients, the likelihood of fathering a biological child increases significantly after mTESE. Without this procedure, the rate of successful reproduction is only approximately 14.5%. Following successful sperm retrieval, it increases to 32.41%, and with the subsequent assessment of sperm suitability for ICSI cycles, the success rate reaches 46.82% (13). In recent years, mTESE-ICSI has been widely adopted for NOA patients, significantly improving fertilization and pregnancy rates. Previous research on patients with AZF deletions undergoing mTESE-ICSI has revealed variations in sperm utilization between synchronous and asynchronous procedures, but no significant differences were found in the SRR or ICSI success rates (14). These results suggest that synchronous surgical strategies may offer advantages in terms of sperm utilization and cumulative live birth rates for AZFc-deleted NOA patients. When comparing fresh and cryopreserved testicular sperm obtained via mTESE-ICSI, the main difference between synchronous and asynchronous procedures is the “freshness” of the sperm, which could influence the pregnancy and live birth rates. While there were no notable differences in patient characteristics, fertilization rates, or the rates of good-quality embryos between the two groups, existing evidence suggests that fresh sperm may provide better pregnancy and live birth outcomes than cryopreserved sperm does (15). Numerous studies have indicated that there is no notable difference in clinical outcomes between the use of cryopreserved and fresh testicular sperm for ICSI (16). However, Zhihong Zhang reported a higher miscarriage rate and a lower live birth rate when cryopreserved sperm was utilized (17). In addition, research by Madureira highlighted that fresh sperm obtained via mTESE from nonmosaic KS patients led to better fertilization and clinical pregnancy outcomes (18). Cryopreservation plays a crucial role in reducing unnecessary ovarian stimulation in women and minimizing the need for repeated testicular biopsies during ICSI cycles (17). It has shown benefits for NOA patients and those with small testes (19). Moreover, ensuring sperm availability during ART cycles is vital for ensuring the safety and efficiency of these procedures (20, 21). However, some studies have raised concerns that cryopreservation could adversely affect sperm quality, potentially reducing ART success rates (22, 23). The debate on the best practices for cryopreserving testicular sperm remains unresolved. Previous studies have predominantly compared synchronous and asynchronous procedures in NOA patients, often with small sample sizes and without stratified analysis. In contrast, our research specifically focused on KS patients and included a large-scale study comparing fresh versus cryopreserved sperm in this population to evaluate ICSI treatment outcomes. Our study revealed that the pregnancy outcomes were largely consistent with those reported in the previous studies, with no significant differences. However, in terms of laboratory metrics, synchronous group presented greater averages of oocyte retrieval, MII oocytes, gestational sacs, and good-quality embryos than did asynchronous group. These findings highlight a statistically significant difference in reproductive outcomes between fresh and cryopreserved sperm, with fresh sperm demonstrating an advantage in improving embryo quality. We subsequently performed LASSO regression and receiver operating characteristic (ROC) curve analysis for model prediction and found that these differential indicators have limited ability to predict pregnancy outcomes. These findings further support the conclusion that there is no significant difference in pregnancy outcomes between synchronous and asynchronous surgeries. This discovery provides valuable clinical guidance for assisted reproductive treatments in KS patients. Despite these valuable insights, our study has several limitations. The retrospective design and single-center data limit the generalizability of the results. Moreover, the lack of long-term follow-up after delivery means that we were unable to assess whether cryopreserved sperm impact neonatal outcomes or the long-term development of offspring. Future prospective studies with multicenter data are necessary to further explore the effects of cryopreservation on pregnancy outcomes, particularly in terms of the long-term health of the offspring.

## 5 Conclusions

Our study revealed no significant difference in pregnancy outcomes between KS patients who underwent mTESE-ICSI with fresh testicular sperm and those with cryopreserved sperm. Therefore, we can conclude that there is no difference in the impact of synchronous versus asynchronous surgery on pregnancy outcomes. However, multicenter prospective studies are needed in the future to systematically evaluate the clinical efficacy of fresh versus cryopreserved sperm in patients with KS. With the development of personalized treatment protocols and the accumulation of clinical data, fertility treatments for KS patients will become more precise, ultimately improving pregnancy success rates and quality of life for these patients.

## Data Availability

The original contributions presented in the study are included in the article/supplementary material. Further inquiries can be directed to the corresponding author.
